# Fucosylated Glycosaminoglycan Oligosaccharide HS14, Derived from Sea Cucumbers, Is a Novel Inhibitor of Platelet Toll-like Receptor 2

**DOI:** 10.3390/md23030110

**Published:** 2025-03-04

**Authors:** Huifang Sun, Guangyu Zhu, Sujuan Li, Pengfei Li, Jiali Zhang, Ronghua Yin, Lin Yuan, Na Gao, Jinhua Zhao

**Affiliations:** 1 School of Chemistry and Materials Science, South-Central Minzu University, Wuhan 430074, China; sunhuifang2021@outlook.com; 2 College of Life Sciences, South-Central Minzu University, Wuhan 430074, China; 15347008757@163.com; 3 State Key Laboratory of Phytochemistry and Plant Resources in West China, Kunming Institute of Botany, Chinese Academy of Sciences, Kunming 650201, China; lisujuan1988@126.com; 4 School of Pharmaceutical Sciences, South-Central Minzu University, Wuhan 430074, China; 13708322361@163.com (P.L.); zhangjiali20221123@163.com (J.Z.); yinrh77@163.com (R.Y.); yuanlinjx@126.com (L.Y.)

**Keywords:** toll-like receptor 2, platelet activation, platelet aggregation, platelet–granulocyte aggregates, fucosylated glycosaminoglycan, inflammatory diseases

## Abstract

(1) Background: Toll-like receptor 2 (TLR2) on platelets is increasingly recognized as a pivotal mediator in infection-induced platelet activation and aggregation, contributing to both inflammatory and thrombotic diseases. Targeting TLR2 on platelets offers a promising therapeutic strategy for inflammatory and thrombotic-related disorders. However, inhibitors targeting platelet TLR2 have not yet been reported. (2) Methods: Platelet aggregation was assessed using a light transmission aggregometer. Platelet activation was evaluated by measuring the release of P-selectin and von Willebrand factor (vWF) via ELISA. Intracellular Ca^2+^ mobilization was quantified using Fluo 3-AM fluorescence, recorded by flow cytometry. Static platelet adhesion was visualized under a microscope, and the formation of platelet–granulocyte aggregates in human whole blood was analyzed by flow cytometry. (3) Results: Fucosylated glycosaminoglycan (FG) tetradecasaccharide HS14 inhibited the activation and aggregation of human platelets induced by the synthetic bacterial lipopeptide Pam3CSK4 in a concentration-dependent manner. This inhibitory effect gives rise to significant anti-inflammatory and anti-thrombotic activities, as evidenced by reduced platelet adhesion and decreased platelet–granulocyte aggregates formation in human whole blood. (4) Conclusions: This study is the first to identify FG oligosaccharide HS14 as a promising inhibitor of platelet TLR2/TLR1, demonstrating significant therapeutic potential for inflammatory and thrombotic-related diseases.

## 1. Introduction

Platelets are anucleate blood cell fragments primarily derived from megakaryocytes in the bone marrow. They possess a complete plasma membrane, with a diameter ranging from 2 to 3 μm, and a lifespan of approximately 8 to 10 days in blood circulation [1]. The platelet count in human blood is roughly 40 to 50 folds higher than that of leukocytes. Initially, platelets were recognized for their role in hemostasis and promoting blood coagulation [2]. However, over the past two decades, the immunoregulatory functions of platelets and their roles in related diseases have received extensive attention [3,4]. Accumulating evidence suggests that upon the entry of microbial or non-microbial antigens into the bloodstream, platelets can function as frontline sensing cells, exerting innate or adaptive immune responses [5,6,7]. The immune functions of platelets are mediated by pattern recognition receptors such as Toll-like receptors (TLRs), NOD-like receptors, and C-type lectin receptors, which play crucial roles in detecting and responding to pathogen-associated molecular patterns (PAMPs) and damage-associated molecular patterns [8]. Consequently, platelets serve not only as the primary mediators of hemostasis but also as key inflammatory effector cells, playing crucial roles in hemostatic, thrombotic, and immune–inflammatory processes [9].

TLRs are innate immune receptors that mediate cellular immune responses and inflammatory reactions by recognizing PAMPs [10]. Human platelets express various functional TLRs, including TLR2, TLR4, and TLR9, which play crucial roles in thrombosis [11,12]. Specifically, TLR2 is located on the cell membrane and recognizes a broad range of PAMPs through heterodimerization with either TLR1 or TLR6 [13]. A substantial body of research has demonstrated that platelet TLR2 plays a vital role in platelet activation, aggregation, and thrombosis promotion. In 2009, Blair et al. [14] first reported that stimulating TLR2 can induce platelet activation and increase TLR2 expression on the platelet surface, thereby confirming that platelet TLR2 functions as a receptor involved in both thrombotic and inflammatory responses. Furthermore, the study by Keane et al. [15] demonstrates that invasive *Streptococcus pneumoniae* induces platelet activation via TLR2, potentially resulting in thrombotic complications associated with sepsis. Pam3CSK4, a synthetic bacterial lipopeptide and potent agonist of the TLR2/TLR1 heterodimer, predominantly interacts with TLR2 and is widely used as a ligand for investigating TLR2 function [16]. This compound can induce human platelet cytosolic calcium (Ca^2+^) mobilization, dense granule secretion, and platelet aggregation through activating Src family tyrosine kinases [17].

Platelet TLR2 plays important roles in multiple diseases, including infection, thrombosis, inflammation, autoimmunity, cancer, neurodegenerative disorders, and metabolic conditions. Among these, sepsis—a condition marked by high incidence, significant disability, and elevated mortality—deserves particular attention. Sepsis is defined as a systemic inflammatory response syndrome resulting from infection [18]. And it has been demonstrated that platelets play a crucial role in the pathophysiology of sepsis, as uncontrolled bacterial infections trigger extensive platelet activation, leading to thrombocytopenia [19]. The extent of thrombocytopenia is closely associated with the severity of sepsis. Given that bacteria can directly induce platelet activation via TLR2 [15,20], inhibiting this pathway may be a potential strategy to mitigate or delay the progression of sepsis. In 2020, Morgan et al. [21] proposed that TLR2 serves as a potential therapeutic target in bacterial infections. To date, only a limited number of inhibitors targeting TLR2 or its signaling pathways have been reported [22,23,24,25,26], and whether these inhibitors can effectively inhibit human platelet activation and aggregation triggered by TLR2 remains unexplored.

Native fucosylated glycosaminoglycan (FG) derived from sea cucumbers, consisting of a chondroitin sulfate-like backbone and unique sulfated fucose-containing branches, exhibits a wide range of pharmacological activities, including anti-tumor, anti-virus, anticoagulation, and anti-thrombosis effects [27,28]. Among these, its anticoagulant and anti-thrombotic properties have been the most extensively studied [29]. Significantly, the FG derivative dHG-5 is being developed as a novel anticoagulant drug. It has received approval from both the US Food and Drug Administration and the National Medical Products Administration of China and is currently undergoing Phase I clinical trials (CTR20223258, http://www.chinadrugtrials.org.cn/clinicaltrials.searchlist.dhtml (accessed on 19 December 2022)). dHG-5 is a multicomponent entity comprising a series of oligosaccharides, which is generated via controlled β-eliminative depolymerization of FG from the sea cucumber *Holothuria fuscopunctata* [30]. These oligosaccharides, which constitute dHG-5, have been purified and their structures have been thoroughly characterized, providing a solid material foundation for investigating their pharmacological activities. In 2022, Li et al. [31] first demonstrated that FG oligosaccharides can inhibit thrombin-induced platelet activation and aggregation, offering novel insights in exploring the pharmacological activities of FG oligosaccharides.

**Figure 1:**
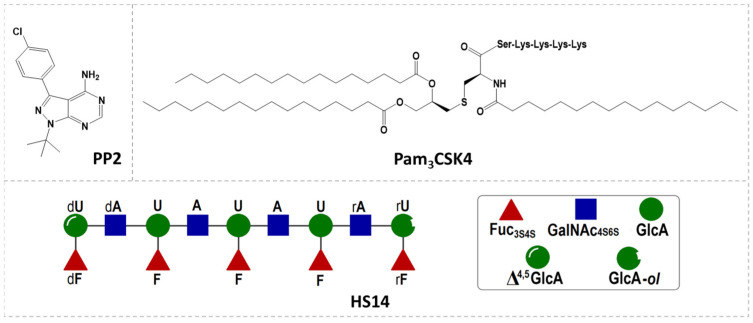
Structures of HS14, Pam3CSK4, and PP2. HS14—tetradecasaccharide derived from sea cucumber *Holothuria fuscopunctata*; Pam3CSK4—synthetic bacterial lipopeptide and potent agonist of TLR2/TLR1 heterodimer; PP2—selective inhibitor of Src family tyrosine kinases.

Whether to explore the pharmacological activities of FG oligosaccharides or to address the urgent need for platelet TLR2 inhibitors, investigating the effects of FG oligosaccharides on platelet TLR2 holds significant importance. In this study, we investigated the effect of tetradecasaccharide HS14 (a representative oligosaccharide in dHG-5, with a degree of polymerization of 14 and Mw of 4373 Da) on Pam3CSK4-induced human platelet responses (Figure 1). Excitingly, HS14 demonstrated a concentration-dependent suppression of human platelet activation and aggregation triggered by the synthetic bacterial lipopeptide Pam3CSK4. This suppression was associated with notable anti-inflammatory and anti-thrombotic effects, as shown by diminished platelet adhesion and the reduced formation of platelet–granulocyte aggregates in human whole blood. This is the first report demonstrating that FG oligosaccharide can inhibit Pam3CSK4-mediated platelet reactions, which may provide a novel therapeutic opportunity for inflammatory and thrombotic-related diseases, especially sepsis.

## 2. Results

### 2.1. HS14 Inhibited Pam3CSK4-Induced Human Platelet Aggregation and α-Granule Secretion

Although platelet aggregation is crucial for physiological hemostasis, under specific pathological conditions, including atherosclerosis, diabetes, and hypertension, excessive platelet aggregation can contribute to thrombosis, thereby increasing the risk of severe conditions such as myocardial infarction and stroke. Previous studies have demonstrated that Pam3CSK4 (a synthetic triacylated bacterial lipopeptide known to interact with TLR2/TLR1 heterodimers) could potently induce human platelet aggregation, resulting in a thromboinflammatory response [14]. However, whether FG derived from sea cucumbers, consisting of a chondroitin sulfate-like backbone and unique sulfated fucose-containing branches, can inhibit this process has not been investigated yet. To determine this, HS14, a representative oligosaccharide in dHG-5, with a degree of polymerization of 14 and Mw of 4373 Da, was added to stirred platelet suspensions, and Pam3CSK4 was used for inducing human platelet aggregation.

**Figure 2:**
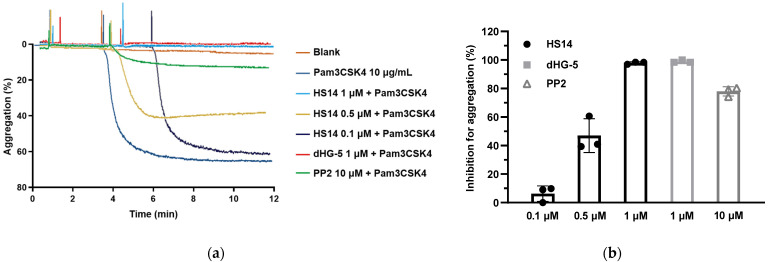
HS14 inhibited Pam3CSK4-induced human platelet aggregation. (**a**) Human platelets were incubated with inhibitor or vehicle at 37 °C for 3 min under stirring condition (1000 revolutions per minute), and then Pam3CSK4 (10 μg/mL) was added to induce platelet aggregation. Untreated platelets were included as blank control. The shown data are from one of three individual experiments. (**b**) Inhibition rate of inhibitors for Pam3CSK4-induced human platelet aggregation. Data of three individual experiments are presented as mean ± standard deviation (SD). dHG-5—β-eliminative depolymerization product with average Mw of about 5.2 kDa, obtained from sea cucumber *Holothuria fuscopunctata*.

In our study, Pam3CSK4 at 10 μg/mL was sufficient to elicit full human platelet aggregation. Thus, this concentration of Pam3CSK4 was used for subsequent assays. Surprisingly, HS14 inhibited Pam3CSK4-induced human platelet aggregation in a concentration-dependent manner, with inhibition rates of 97.94 ± 0.84% at 1 μM, 46.99 ± 11.86% at 0.5 μM, and 6.31 ± 5.48% at 0.1 μM (Figure 2). To verify the inhibitory activity, dHG-5 and PP2 were used in subsequent assays. dHG-5 is a multicomponent entity from the sea cucumber *Holothuria fuscopunctata* with an Mw of 5.2 kDa. PP2 is a selective inhibitor of Src family tyrosine kinases (Figure 1). Previous studies have demonstrated that PP2 effectively inhibits Pam3CSK4-induced cytosolic calcium (Ca^2+^) mobilization, dense granule secretion, and platelet aggregation in human platelets by blocking the activation of Src family tyrosine kinases [32]. As expected, both dHG-5 and PP2 nearly completely inhibited Pam3CSK4-induced human platelet aggregation at the tested concentrations (Figure 2).

The release of platelet α-granules is a critical component of platelet function, essential for maintaining normal physiological hemostasis, vascular repair, and inflammatory responses. However, aberrant α-granule release can contribute to thrombotic and inflammatory diseases [33]. In conditions such as atherosclerosis, myocardial infarction, and stroke, excessive platelet activation results in the over-release of α-granules. Procoagulant factors within these granules, including platelet factor 4 (PF4), β-thromboglobulin, and fibrinogen, accelerate thrombus formation, thereby exacerbating vascular occlusion and tissue ischemia. During infection, inflammatory mediators (e.g., PF4, RANTES) and chemokines released from α-granules promote leukocyte infiltration and inflammation, contributing to the pathogenesis of inflammatory diseases like atherosclerosis and rheumatoid arthritis.

**Figure 3:**
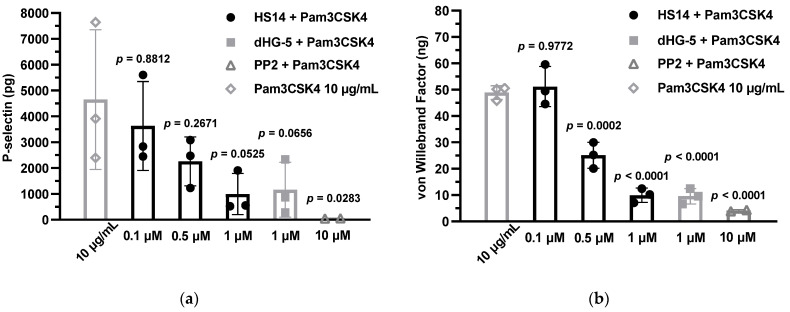
HS14 inhibited Pam3CSK4 (10 μg/mL)-induced human platelet α-granule secretion. After the platelet aggregation test was completed, the samples were centrifuged first at 1100× *g* for 5 min, followed by 9300× *g* for 5 min, and the supernatants were stored at −80 °C until assayed. The contents of P-selectin (**a**) and vWF (**b**) in the supernatants were determined by ELISA. Data of two or three individual experiments are presented as mean ± SD. Statistical significance was determined by comparing the results to those of the Pam3CSK4 group.

Because previous studies have demonstrated that Pam3CSK4 can induce the release of granule contents in human platelets [14], we investigated whether HS14 could inhibit platelet α-granule release triggered by Pam3CSK4. For this purpose, P-selectin and vWF in the platelet supernatants were measured as markers of α-granule release. Consistent with observations in platelet aggregation experiments, Pam3CSK4 (10 μg/mL) induced the secretion of both P-selectin and vWF in human platelets. The presence of HS14 and dHG-5 at 1 μM significantly inhibited the secretion of vWF (*p* < 0.0001). While HS14 and dHG-5 at 1 μM also inhibited the secretion of P-selectin, this inhibition was not statistically significant (*p* > 0.05). Notably, HS14 exhibited a concentration-dependent (applied from 0.1 up to 1 μM) inhibition of Pam3CSK4-induced P-selectin and vWF release from human platelets (Figure 3). Additionally, PP2 completely inhibited Pam3CSK4-induced P-selectin and vWF release, further supporting the role of Src kinases as mediators in α-granule content release in response to TLR2 activation [17].

### 2.2. HS14 Inhibited Pam3CSK4-Induced Human Platelet Cytosolic Ca^2+^ Mobilization

**Figure 4:**
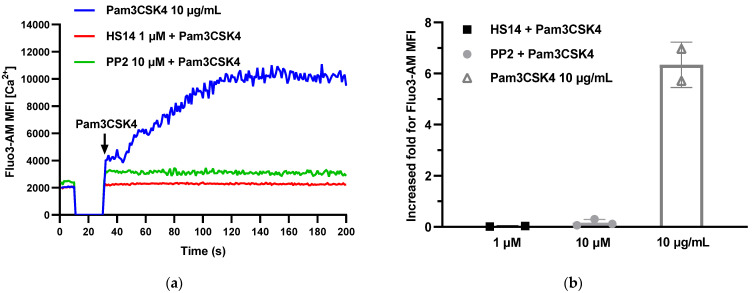
HS14 terminated human platelet cytosolic Ca^2+^ mobilization induced by Pam3CSK4 (10 μg/mL). (**a**) This plot shows the integral of the intra-platelet fluorescence intensity at different time points. Pam3CSK4 (10 μg/mL) was added at the 30 s. The shown data are from one of three individual experiments. (**b**) Maximal peak rises in human platelet cytosolic Ca^2+^ under indicated conditions were determined as described in Section 4. Data of two or three individual experiments are presented as mean ± SD.

The mobilization of cytoplasmic Ca^2+^ in human platelets is a pivotal event in platelet activation, aggregation, and secretion [34]. Upon stimulation by agents such as collagen, thrombin, or adenosine diphosphate (ADP), the intracellular Ca^2+^ concentration increases rapidly, thereby inducing morphological changes, aggregation, and secretion in platelets. This Ca^2+^ mobilization triggers the release of α-granules and dense granules from platelets, which contain substances like ADP, 5-hydroxytryptamine, and coagulation factors. These released substances further amplify the processes of platelet activation and aggregation. Moreover, the Ca^2+^ signal plays a crucial role in mediating interactions between platelets and leukocytes as well as endothelial cells, thereby influencing inflammatory and immune responses. Previous studies demonstrated that the secretion and aggregation of human platelets induced by Pam3CSK4 are mediated through intracellular Ca^2+^ mobilization [35]. We further investigated the effect of HS14 on Pam3CSK4-induced cytosolic Ca^2+^ mobilization in human platelets. As shown in Figure 4, treatment with Pam3CSK4 (10 μg/mL) resulted in a sixfold increase in cytosolic Ca^2+^ levels in human platelets. Notably, both HS14 (1 μM) and PP2 (10 μM) completely inhibited Pam3CSK4-induced cytosolic Ca^2+^ mobilization. These results further confirm that HS14 can effectively inhibit the activation and aggregation of human platelets induced by Pam3CSK4.

### 2.3. HS14 Inhibited Pam3CSK4-Induced Static Human Platelet Adhesion

**Figure 5:**
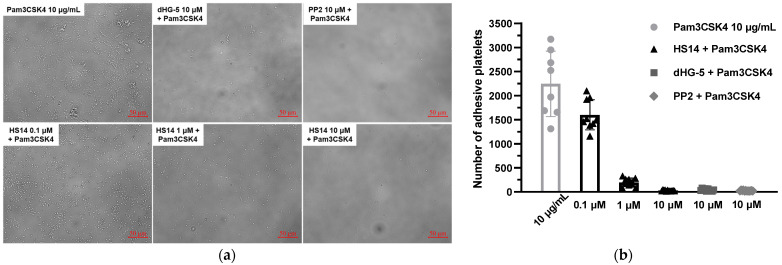
HS14 inhibited Pam3CSK4-induced human platelet adhesion to bovine serum albumin (BSA). Human platelets were treated with vehicle and inhibitors before stimulation with Pam3CSK4 (10 μg/mL) for 1 h in BSA-coated 96-well microplates. After washing, the adhesive platelets were visualized using a microscope (**a**). The shown pictures are from one of three individual experiments. The number of adhesive platelets was analyzed (**b**). The experiments were independently replicated three times, with 2–3 fields of view analyzed for each replicate. Data are presented as mean ± SD.

Platelet adhesion serves as the essential initiating factor in acute thrombotic events, including myocardial infarction, stroke, and deep vein thrombosis [36]. Platelets monitor the endothelial cell layer of the vasculature for injuries and become activated upon adherence to subendothelial structures, including bacterial components. This activation leads to the recruitment of leukocytes through cytokine and chemokine secretion, thereby initiating inflammation. This process contributes to the pathogenesis of diseases like atherosclerosis and rheumatoid arthritis. In certain infections, such as sepsis, excessive platelet adhesion can result in microvascular thrombosis and multiple organ dysfunction. In this study, we investigated the effect of HS14 on Pam3CSK4-induced static human platelet adhesion. The number of adhered platelets is presented in Figure 5. As anticipated, HS14 demonstrated a concentration-dependent inhibition of Pam3CSK4-induced static human platelet adhesion, with inhibition rates of 98.79 ± 0.41% at 10 μM, 91.19 ± 3.98% at 1 μM, and 28.67 ± 14.02% at 0.1 μM. Notably, both HS14 and dHG-5 almost completely inhibited Pam3CSK4-induced static human platelet adhesion at 10 μM (Figure 5). Additionally, PP2 (10 μM) fully inhibited Pam3CSK4-induced static human platelet adhesion, which aligns with previous findings that Src and Syk kinases are involved in TLR2/TLR1-mediated platelet adhesion [32].

### 2.4. HS14 Inhibited Pam3CSK4-Induced Formation of Platelet–Granulocyte Aggregates in Human Whole Blood

The formation of platelet–granulocyte aggregates (PGAs) plays a critical role in various pathological processes, including inflammation, thrombosis, and infection [37]. PGAs enhance the activation and migration capabilities of neutrophils, thereby promoting the release of inflammatory mediators such as cytokines, chemokines, and reactive oxygen species. This exacerbates the inflammatory response and contributes significantly to chronic inflammatory diseases like rheumatoid arthritis, inflammatory bowel disease, and asthma. Additionally, PGAs facilitate thrombosis by intensifying the interaction between platelets and neutrophils, leading to increased vascular occlusion and tissue ischemia in conditions such as atherosclerosis, myocardial infarction, and stroke. In severe infections, such as sepsis and disseminated intravascular coagulation, excessive PGA formation can trigger cytokine storms and cause tissue damage, resulting in microvascular thrombosis and multiple organ dysfunction.

**Figure 6:**
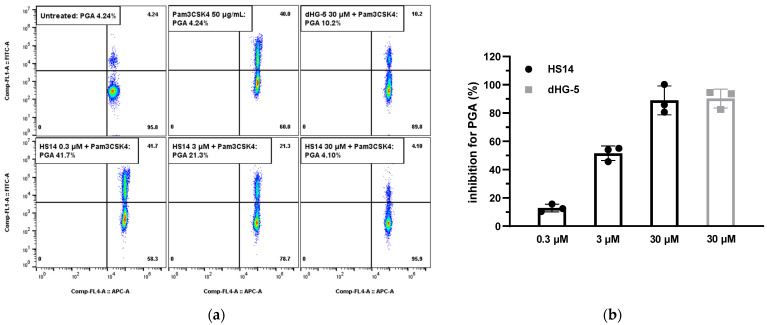
HS14 inhibited Pam3CSK4-induced formation of platelet–granulocyte aggregates (PGAs) in human whole blood. (**a**) Human whole blood was treated with vehicle and inhibitors before stimulation with Pam3CSK4 (50 μg/mL). After lysing red blood cells, samples were analyzed by flow cytometry. Granulocytes were labeled with APC anti-human CD45 antibody, platelets were labeled with FITC anti-human CD61 antibody, and platelet–granulocyte aggregates (PGAs) were identified by simultaneous expression of both FITC and APC. Shown data are from one of three individual experiments. (**b**) Inhibition rate of inhibitors for Pam3CSK4-induced PGAs. Data of three individual experiments are presented as mean ± SD.

It has been demonstrated that Pam3CSK4 can induce the formation of platelet–leukocyte aggregates in human whole blood [14]. Therefore, we investigated the effect of HS14 on Pam3CSK4-induced platelet–leukocyte aggregates in human whole blood. Flow cytometric analysis of whole blood samples confirmed that Pam3CSK4 (50 μg/mL) significantly stimulated the formation of platelet–granulocyte aggregates compared to untreated controls (Figure 6a). The generation of platelet–neutrophil aggregates was significantly inhibited by pretreatment with HS14 and dHG-5 at 30 μM. The half-maximal inhibitory concentration (IC_50_) value was approximately 3 μM. Consistent with its effects on platelet aggregation, secretion, and adhesion, HS14 exhibited a concentration-dependent inhibition of Pam3CSK4-induced platelet–granulocyte aggregate formation in human whole blood, with concentrations ranging from 0.3 to 30 μM. Higher concentrations of HS14 and dHG-5 were required to inhibit the formation of platelet–granulocyte aggregates compared to those used in platelet aggregation experiments, likely due to the presence of additional TLR-expressing cells in whole blood.

## 3. Discussion

TLR2, as a pivotal member of the TLR family, is extensively expressed in both immune and non-immune cells. Initially, research predominantly centered on the role of TLR2 in immune cells, particularly macrophages and dendritic cells. However, as studies advanced, it became evident that platelets also express TLR2. Platelet TLR2 not only contributes to hemostasis and thrombosis but also plays a crucial role in infection, inflammation, and immune regulation. Through TLR2, platelets recognize pathogens such as bacteria and viruses, and upon activation, release inflammatory mediators like chemokines and cytokines to recruit immune cells to the site of infection [38]. Moreover, platelet TLR2 enhances the immune response by interacting with neutrophils and monocytes [39]. Following TLR2 activation, platelets secrete pro-inflammatory factors and procoagulant substances, thereby promoting thrombosis. This process is critical in infection- or inflammation-associated thrombotic diseases, including sepsis and atherosclerosis [40].

Given the critical role of TLR2 in various diseases, developing TLR2 inhibitors has emerged as a promising strategy for treating related conditions. The small molecule inhibitor CU-CPT22 has demonstrated significant anti-inflammatory effects in animal models of sepsis and inflammatory diseases [41]. Similarly, C29 has shown promising therapeutic efficacy in animal models of atherosclerosis and rheumatoid arthritis [42]. The humanized anti-TLR2 monoclonal antibody OPN-305 has exhibited favorable safety and efficacy profiles in clinical trials for sepsis and ischemia–reperfusion injury [43]. Additionally, certain natural compounds, such as curcumin and resveratrol, have been identified as potential TLR2 inhibitors. Possibly through multi-target mechanisms, curcumin has displayed robust anti-inflammatory and immunomodulatory activities in animal models of inflammatory diseases and cancer [44]. Similarly, resveratrol has also shown substantial therapeutic benefits in animal models of atherosclerosis and metabolic disorders [45]. Given the significant blocking effects of HS14 and dHG-5 on platelet TLR2, these compounds may possess considerable potential for therapeutic applications in conditions such as sepsis, atherosclerosis, and rheumatoid arthritis.

**Figure 7:**
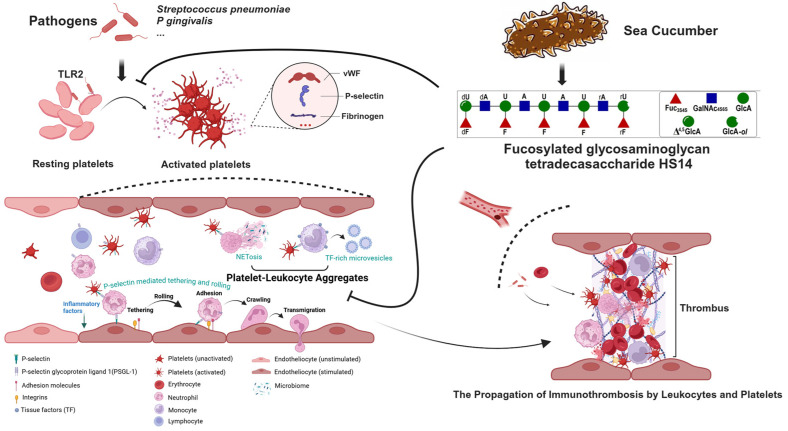
Inhibition of fucosylated glycosaminoglycan tetradecasaccharide HS14, derived from sea cucumber *Holothuria fuscopunctata*, on human platelet TLR2. Following TLR2 activation, platelets become activated, thereby secreting pro-inflammatory cytokines and procoagulant molecules and promoting the formation of platelet–leukocyte aggregates. This process plays a crucial role in infection- or inflammation-associated thrombotic diseases (created with BioRender.com).

FG, a sulfated polysaccharide derived from sea cucumbers, possesses a unique chemical structure and exhibits extensive biological activities, making it highly promising for applications in medicine, food, and cosmetics. However, the complex extraction and purification process of FG oligosaccharides has limited its research and development. Building on the solid foundation of our team’s early work on FG chemistry and pharmacology, we have the opportunity to further explore the pharmacological activities of FG oligosaccharides. In this study, we first demonstrated that fucosylated glycosaminoglycan (FG), derived from sea cucumbers, acts as a novel and potent inhibitor of platelet TLR2/TLR1. Our findings reveal that FG can effectively block the activation and aggregation of human platelets induced by the synthetic bacterial lipopeptide Pam3CSK4. This inhibitory effect translates into significant anti-inflammatory and anti-thrombosis activities, as evidenced by the reduced platelet adhesion and platelet–granulocyte aggregates in human whole blood (Figure 7). These findings may provide novel insights into the anti-thrombotic and anti-inflammatory mechanisms of FG, offer new strategies for the treatment of infection- or inflammation-associated thrombotic diseases, especially sepsis, and open up a novel research direction for the pharmacological activities of FG.

This paper presents an initial investigation into the blocking activity of FG oligosaccharides on platelet TLR2. However, several questions remain to be addressed. Firstly, TLR2 forms heterodimers with TLR1 or TLR6, which complicates signal transduction pathways. Moreover, TLR2 shares structural and functional similarities with other TLRs (e.g., TLR1, TLR4, and TLR6) [46], necessitating further exploration of the selectivity of FG oligosaccharides for these targets. Secondly, the biological functions of platelet TLR2 and its role in various diseases are not yet fully understood, underscoring the need to elucidate the pharmacological activities of FG oligosaccharides in different disease models. Additionally, the precise molecular mechanisms underlying the interaction between FG oligosaccharides and TLR2/TLR1, including the identification of specific binding sites, warrant further investigations.

In conclusion, our study is the first to identify FG oligosaccharides as a promising natural TLR2/TLR1 inhibitor with significant therapeutic potential for inflammatory and immune-related diseases. Future research should focus on comprehensively evaluating the efficacy and structure–activity relationship of FG oligosaccharides in platelet TLR2-mediated diseases, thereby advancing its development toward clinical applications. This work not only expands our understanding of marine-derived polysaccharides but also opens new avenues for the development of TLR2-targeted therapeutics.

## 4. Materials and Methods

### 4.1. Reagents

PP2 (4-amino-5-(4-chlorophenyl)-7-(t-butyl)pyrazolo [3,4-d]pyrimidine), a selective inhibitor of Src family tyrosine kinases, was purchased from Sigma-Aldrich (Taufkirchen, Germany). Pam3CSK4 (Pam3CysSerLys4), a synthetic triacylated lipopeptide (LP) and a TLR2/TLR1 ligand, was purchased from InvivoGen (Toulouse, France). Enzyme-linked immunosorbent assay (ELISA) kits for P-selectin (SELP) and vWF were kindly provided by Cloud-Clone Corp. (Wuhan, China). Fluo 3-AM (Fluo-3-pentaacetoxymethyl ester), a fluorescent Ca^2+^ chelator, was purchased from MedChemExpress (Monmouth Junction, NJ, USA). Fluorescein Isothiocyanate (FITC) anti-human CD61 antibody and Allophycocyanin (APC) anti-human CD45 antibody were purchased from BioLegend (San Diego, CA, USA). FACS lysing solution was purchased from BD bioscience (Franklin Lakes, NJ, USA). Bovine serum albumin (BSA) was purchased from BioVision Technology (Milpitas, CA, USA). PBS Tablets were purchased from MP Biomedicals (Illkirch-Graffenstaden, France). All other chemical reagents were commercially available and of analytical grade.

### 4.2. Preparation of dHG-5 and HS14 [30,47]

dHG-5 was previously prepared from natural fucoidan (FG) extracted from dried sea cucumber *Holothuria fuscopunctata* using β-eliminative depolymerization. This process involved digesting the dried body wall with papain, releasing polysaccharides with 0.25 M sodium hydroxide, and purifying the crude polysaccharide through repeated salting-out with KOAc and ethanol precipitation, followed by strong anion-exchange chromatography using FPA98 resin (with a yield of ~0.8%). Native FG was then chemically cleaved to produce low-molecular-weight fragments via β-eliminative cleavage of its activated benzyl ester derivative. FG was dissolved in distilled water, transalkylated with benzethonium salts, precipitated, and dried under vacuum. The FG benzethonium salts were esterified with benzyl chloride in DMF at 35 °C for 24 h, cooled to 25 °C, and depolymerized by adding freshly prepared 0.08 M EtONa in ethanol. Sodium chloride and ethanol were added sequentially to complete transalkylation to sodium salts. Alkaline saponification was performed to hydrolyze residual benzyl esters, and reducing ends were converted to alcoholic hydroxyl groups using NaBH4. The depolymerized product was isolated by tangential flow filtration using a Pellicon Mini device with a PLCTK membrane (3 kDa or 10 kDa cutoff). Fractions with Mw between 3 kDa and 10 kDa were collected and freeze-dried to obtain dHG-5. The purification of HS14 (tetradecasaccharide) from dHG-5 was previously performed using gel permeation chromatography on Bio-Gel P10 columns. dHG-5 was dissolved in deionized water and applied to a Bio-Gel P10 column equilibrated with 0.2 M NaCl and then eluted with the same solution at a flow rate of 12 mL/h. Fractions (2 mL/tube) were collected and absorbance measured at 234 nm. Oligosaccharide fractions were desalted using a Sephadex G-10 column and freeze-dried to obtain HS14.

According to our previous study, native FG had a backbone consisting of repeated {4)-D-glucuronic acid (GlcA)-β(1,3)-*N*-acetyl-D-galactosamine (GalNAc)-β(1,} disaccharide units, and abundant α-L-fucose sulfate (FucS) branches linked to the C3 of each GlcA residue in the backbone. The types of FucS branches in native FG were L-fucose-3,4-disulfates (Fuc_3S4S_, 85%), L-fucose-2,4-disulfates (Fuc_2S4S_, 10%), and L-fucose-4-sulfates (Fuc_4S_, 5%). The molar percentages of the main oligosaccharides with degrees of polymerization of 5, 8, 11, 14, 17, 20, 23, 26, 29, and >29 in dHG-5 were estimated as 4.86, 17.39, 17.55, 15.93, 13.62, 11.22, 6.62, 4.74, 2.95, and 5.12%, respectively, according to the proportion of peak area in HPGPC [30,47]. The structure of HS14 was defined as L-Fuc_3S4S_-α(1,3)-L-Δ^4,5^GlcA-α(1,3)-{D-GalNAc_4S6S_-β(1,4)-[L-Fuc_3S4S_-α(1,]3)-D-GlcA-β(1,3)-}_3_-D-GalNAc_4S6S_-β(1,4)-[L-Fuc_3S4S_-α(1,]3)-D-GlcA-ol [48].

### 4.3. Preparation of Human Platelets

Blood samples were collected aseptically into a sodium citrate vacutainer tube by venipuncture from healthy donors who had not taken any drugs within the 2 weeks prior to sampling. Platelet-rich plasma (PRP) was obtained by blood sample centrifugation at 180× *g* at 22 °C for 15 min. To avoid leukocyte contamination, only the top 75% of PRP was collected [14]. Platelets were isolated from plasma by a gel filtration method as described in [49]. Ca^2+^-free Tyrode buffer (137 mM NaCl, 2 mM KCl, 1 mM MgCl_2_, 0.34 mM Na_2_HPO_4_, 12 mM NaHCO_3_, 5 mM HEPES, 0.35% BSA, 0.1% glucose, pH 7.35) was used for the elution of platelets from the gel column. Ca^2+^ (1 mM) was added prior to platelet stimulation.

### 4.4. Platelet Aggregation

Platelet aggregation was assayed by light transmission in a Chronolog Model 700 aggregometer (Chronolog, Havertown, PA, USA) [31]. Five hundred microliters of Tyrode buffer was added into the cuvette in the control channel. Then, 250 μL of platelet suspension (2 × 10^8^/mL) was incubated with inhibitors or vehicle (0.02% DMSO or PBS) at 37 °C for 3 min under a stirring condition (1000 revolutions per minute). Then, Pam3CSK4 (10 μg/mL) was added, and changes in light transmission were recorded for 8 min. The column chart analysis was completed using GraphPad Prism v8.0.2 software (GraphPad Software, San Diego, CA, USA). The experiments were independently replicated three times.

### 4.5. Measurement of P-Selectin and von Willebrand Factor (vWF)

After the platelet aggregation assay was completed, samples were centrifuged at 1100× *g* for 5 min, followed by 9300× *g* for 5 min [50]. The resulting supernatants were stored at −80 °C until assayed. The released P-selectin and vWF were quantified using ELISA, following the manufacturer’s instructions. The column chart analysis was completed using GraphPad Prism v8.0.2 software (GraphPad Software, San Diego, CA, USA). The experiments were independently replicated two or three times.

### 4.6. Measurement of Intracellular Ca^2+^ Mobilization

Platelets (1 × 10^8^/mL) were incubated with Fluo 3-AM (1 μM) for 20 min at 37 °C in the dark [51]. The incubated platelets were diluted tenfold and incubated with inhibitors or vehicle (0.02% DMSO or PBS) at 37 °C for 10 min. The baselines of intra-platelet fluorescence intensity at different time points were recorded for approximately 30 s by flow cytometry (C6 plus, BD bioscience, Franklin Lakes, NJ, USA). Then, Pam3CSK4 (10 μg/mL) was added, and changes in fluorescence intensity were recorded for 3 min. The data were analyzed using FlowJo V10 software (FlowJo, LLC, Ashland, OR, USA). The column chart analysis was completed using GraphPad Prism v8.0.2 software (GraphPad Software, San Diego, CA, USA). The experiments were independently replicated two or three times.

### 4.7. Static Platelet Adhesion

The adhesion of platelets was measured as previously described [52]. Briefly, 96-well microplates (Corning, Corning, NY, USA) were coated by the addition of 100 μL/well of 2 mg/mL BSA overnight at 4 °C. The microplates were washed twice with 0.9% NaCl by plate inversion. Then, 100 μL of platelets (5 × 10^7^/mL) was added and incubated with inhibitors or vehicle (0.02% DMSO or PBS) at 37 °C for 10 min. Then, Pam3CSK4 (10 μg/mL) was added, and the microplates were incubated for 1 h at room temperature. After incubation, the microplates were washed twice by plate inversion in 0.9% NaCl. After washing, the adhesive platelets were visualized using the microscope (Axio Observer 7, Zeiss, Oberkochen, Germany), and the number of adhesive platelets was analyzed by ImageJ software (ImageJ 1.53, National Institutes of Health, Bethesda, MD, USA). The column chart analysis was completed using GraphPad Prism v8.0.2 software (GraphPad Software, San Diego, CA, USA). The experiments were independently replicated three times, with 2–3 fields of view analyzed for each replicate.

### 4.8. Analysis of PGA Formation by Flow Cytometer

The blood was diluted three times with PBS. Then, the platelet-specific antibody FITC-anti-CD61 and the leukocyte-specific antibody APC-anti-CD45 were added, following the manufacturer’s instructions. The cocktail was incubated with inhibitors or vehicle (PBS) at 37 °C for 10 min, followed by the addition of Pam3CSK4 (50 μg/mL). The cocktail was incubated at 37 °C for 25 min in the dark. Then, the FACS lysing solution was added to lyse red blood cells for 5 min. After lysis, the cocktail was centrifuged, and the resulting pellet was resuspended in PBS buffer for analysis by flow cytometry (C6 Plus, BD Biosciences, Franklin Lakes, NJ, USA). A minimum of 5000 granulocytes were acquired. The ratio of granulocytes co-expressing CD45 and CD61 to the total granulocyte population was quantified to evaluate the efficacy of the compounds in inhibiting PGA formation [53]. The data were analyzed and visualized using FlowJo V10 software (FlowJo, LLC, Ashland, OR, USA). The column chart analysis was completed using GraphPad Prism v8.0.2 software (GraphPad Software, San Diego, CA, USA). The experiments were independently replicated three times.

### 4.9. Statistical Analysis

Statistical analysis was conducted using GraphPad Prism v8.0.2 software (GraphPad Software, San Diego, CA, USA). The normality of the data was tested through the Shapiro–Wilk test. A one-way analysis of variance followed by Sidak’s multiple comparison test was used for determining mean differences. Statistical significance was defined as a *p* value ≤ 0.05.

## Data Availability

All data generated or analyzed during this study are included in this published article.

## Abbreviations

The following abbreviations are used in this manuscript:

TLR2	Toll-like receptor 2
vWF	von Willebrand factor
FG	fucosylated glycosaminoglycan
TLRs	Toll-like receptors
PAMPs	pathogen-associated molecular patterns
SD	standard deviation
PF4	platelet factor 4
ADP	adenosine diphosphate
PGAs	platelet–granulocyte aggregates
PRP	platelet-rich plasma
